# The impact of microglial activation on blood-brain barrier in brain diseases

**DOI:** 10.3389/fncel.2014.00362

**Published:** 2014-11-03

**Authors:** Anna Carolina Carvalho da Fonseca, Diana Matias, Celina Garcia, Rackele Amaral, Luiz Henrique Geraldo, Catarina Freitas, Flavia Regina Souza Lima

**Affiliations:** Laboratório de Morfogênese Celular, Instituto de Ciências Biomédicas, Centro de Ciências da Saúde, Bloco F, Universidade Federal do Rio de JaneiroRio de Janeiro, RJ, Brazil

**Keywords:** microglia, endothelium, blood-brain barrier, neuroinflammation, brain diseases, microglial activation

## Abstract

The blood-brain barrier (BBB), constituted by an extensive network of endothelial cells (ECs) together with neurons and glial cells, including microglia, forms the neurovascular unit (NVU). The crosstalk between these cells guarantees a proper environment for brain function. In this context, changes in the endothelium-microglia interactions are associated with a variety of inflammation-related diseases in brain, where BBB permeability is compromised. Increasing evidences indicate that activated microglia modulate expression of tight junctions, which are essential for BBB integrity and function. On the other hand, the endothelium can regulate the state of microglial activation. Here, we review recent advances that provide insights into interactions between the microglia and the vascular system in brain diseases such as infectious/inflammatory diseases, epilepsy, ischemic stroke and neurodegenerative disorders.

## Introduction

The cerebrovasculature plays a crucial role in oxygen and nutrient delivery to the organ with the highest metabolic demand in the body, the brain. Moreover, it has a vital function in maintaining a stable ionic environment and protecting against neurotoxic substances critical for normal brain development and function. Brain endothelial cells (ECs) are especially suited for these functions. They act as a selective physical barrier known as the blood brain barrier (BBB), a unique specialized structure of the central nervous system (CNS) capillary bed based on EC tight junctions, with no endothelial fenestrae, and expression of specific membrane transporters, thus ensuring homeostasis and proper functioning of the brain. Brain ECs are closely associated and functionally assembled in what is termed the “neurovascular unit”, composed by ECs, neurons, pericytes and glia (Iadecola, 2004). The neurovascular unit (NVU) is at the basis of neurovascular coupling, which allows cerebral blood flow to be locally regulated according to neuronal activity in specific areas of the brain, contributing to normal CNS functioning (Zlokovic, 2008).

In this review, we discuss current concepts underlying the interactions between the vascular system and glial cells, in particular, the microglia—the CNS resident macrophages—in brain diseases such as infectious/inflammatory diseases, epilepsy, ischemic stroke and neurodegenerative diseases.

## Microglia-endothelium interaction during development

During embryogenesis, the formation of the BBB is a gradual process that starts with sprouting and invagination of newly formed branches from the perineural vascular plexus through angiogenesis (Ruhrberg and Bautch, 2013). At the distal end of the growing capillary lies a specialized EC, known as tip cell (Gerhardt et al., 2003), which guides the vascular sprout by integrating external guidance cues as well as vascular endothelial growth factor (VEGF) gradients in the extracellular matrix (Ruhrberg et al., 2002; Gerhardt et al., 2003). Vascular plexus growth is accomplished by halting tip cell migration, followed by tip cell anastomosis and subsequent lumen formation (Lenard et al., 2013). Finally, vessel stabilization and maturation depends on the association of perivascular cells on the new sprouts (Lindahl et al., 1997). The BBB properties are not intrinsic to CNS ECs, as demonstrated by transplantation experiments using the avian embryo model. Prospective abdominal vessels grafted in contact with neural tissue display functional and histochemical characteristics of the BBB (Stewart and Wiley, 1981). It has been shown that formation of EC tight junctions occurs soon after invasion of the vessels into the developing neuroectoderm (reviewed in Wolburg and Lippoldt, 2002). The ratio of pericyte coverage is highest in the CNS microvessels, which are found to be thoroughly covered by a perivascular astroglial sheath (Mathiisen et al., 2010). Several studies have also shown that components of the NVU such as pericytes and astrocytes play a key role in regulating BBB maintenance and integrity (Janzer and Raff, 1987; Armulik et al., 2010; Daneman et al., 2010). Taken together, these results suggest that the interaction of the ECs with the neural environment is at the basis of the properties of the BBB.

**Figure 1:**
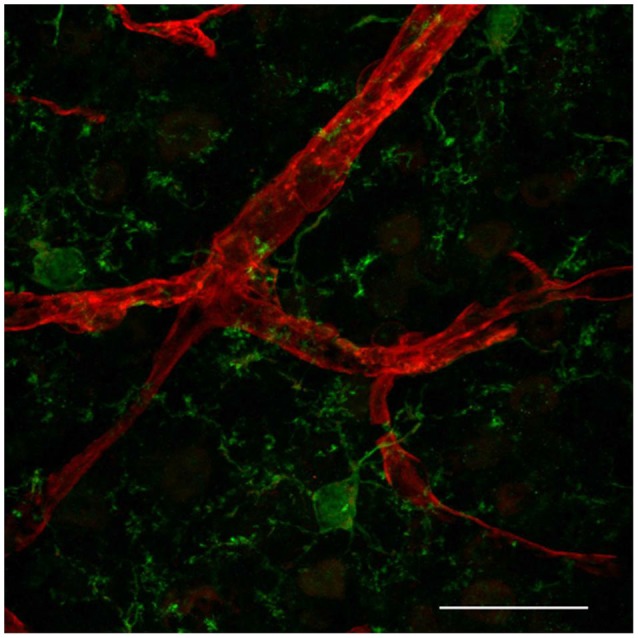
**Resident microglia associate with endothelium in the cortical microvascular bed**. Representative confocal image revealing close interaction between microglia (IBA1, green) and endothelial cells (IsolectinB4, red) in a 50 micron-cryopreserved cross-section of a 1 month-old mouse cortex. Scale bar: 25 µm. This study was approved by the Ethics Committee of the Health Sciences Center at the Federal University of Rio de Janeiro (Protocol No. DAHEICB 015). The “Principles of laboratory animal care” (NIH publication No. 85–23, revised 1996) guidelines as well as The Code of Ethics of EU Directive 2010/63/EU were strictly followed for experiments.

Distinct immune cells within the CNS interact with the BBB. Whereas blood-borne macrophages localize between the vessel wall and the astrocytic endfeet at the meninges, choroid plexus and perivascular spaces (reviewed in Ransohoff and Engelhardt, 2012), recent *in vivo* studies present evidence that resident microglia in the brain parenchyma also interact with CNS microvessels suggesting that, besides an indisputable role in CNS development and homeostasis (reviewed in Nayak et al., 2014), those cells might be also playing a key role in regulating BBB properties during embryogenesis and disease (Fantin et al., 2010; Tammela et al., 2011). Microglia are the most abundant CNS innate immune cells, which during embryogenesis migrate from the yolk sac into the CNS parenchyma (Alliot et al., 1999). Microglia cerebral colonization precedes EC sprouting into this tissue (Cuadros et al., 1993; Checchin et al., 2006; Fantin et al., 2010; Ginhoux et al., 2010) but soon after, they localize in tight physical association with microvascular structures (Fantin et al., 2010; Figure 1), suggesting that microglia may play a role in angiogenesis as well as in conferring BBB properties to brain microvessels. In addition, microglia associate with endothelial tip cells, as demonstrated during embryonic brain and postnatal retinal angiogenesis (Fantin et al., 2010; Rymo et al., 2011; Tammela et al., 2011). Fantin and collaborators present *in vivo* data showing that during embryonic stages of CNS vascularization, EC stabilization and fusion are mediated by resident microglial cells (Fantin et al., 2010). Mice deficient for PU.1 (transcription regulator of CD11b and colony stimulating factor (CSF)-1) have reduced microglia, but not circulating monocytes, and present a decrease in embryonic CNS vascular connections. Because microglia appear to be physically associated with tip cell filopodia and the number of sprouts in not altered, the authors suggest that microglia play a role in CNS angiogenesis by serving as a bridge to promote tip cell fusion, vascular plexus growth following sprouting induction and tip cell migration by VEGF (Fantin et al., 2010). Similarly, specific depletion of microglia using clodronate liposomes (CL2MDP-lip) results in decreased vessel density in a mouse model of choroidal neovascularization (Espinosa-Heidmann et al., 2003). Further *in vivo* evidence for a role of microglia as a cellular chaperone controling the fusion and stabilization of vascular sprouts during CNS vascularization came from the observation that a subpopulation of F4/80/Tie-2 positive cells, specifically located near branching sites at the vascular front during vascularization stages of the retina, express VEGF-C. Despite increased vessel sprouting and filopodia bursts, VEGF-C heterozygotes present delayed retinal vascularization and decreased vessel branching density (Tammela et al., 2011). These F4/80-expressing cells present a ramified morphology, which is typical of microglial cells. The retina is part of the CNS and therefore also presents a proper blood barrier. Since the study by Tammela et al. was performed without any damage to the retinal blood barrier, and resident macrophages of the CNS are microglial cells, it thus constitute further *in vivo* evidence for microglia CNS endothelium interaction during early stages of CNS vascularization.

Although a great deal of literature has demonstrated that microglia play a role in CNS diseases where BBB breakdown is a hallmark, little is known about a possible role of these cells in inducing and/or maintaining BBB properties during CNS EC development. The fact that microglia are present in the embryonic CNS territory prior to endothelial invasion and that they participate in cerebrovasculature growth place the interactions between microglia and CNS endothelium as a possible key mechanism in BBB formation and regulation.

## Microglial activation and the impact on BBB

**Figure 2:**
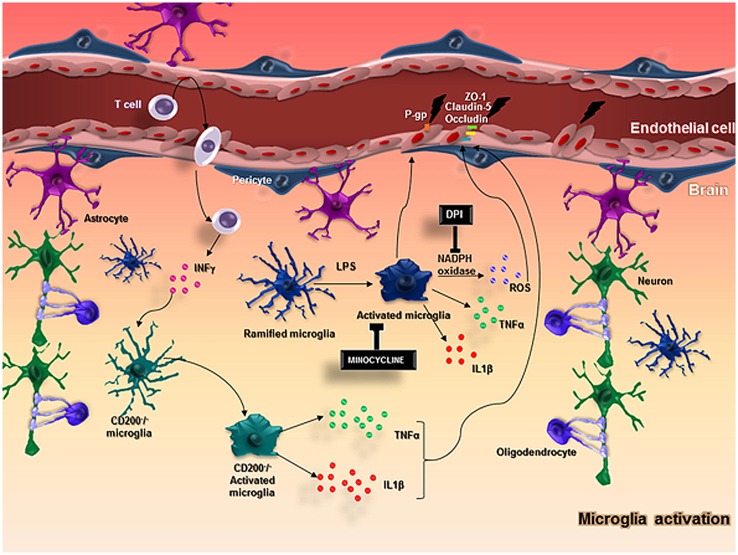
**In normal conditions, neurons and glial cells interact together to promote the homeostasis of the brain**. When an injury occurs in the brain, microglia and astrocytes are capable of producing cytokines and chemokines and stimulate the adhesion molecules on ECs, allowing the migration of myeloid cells from the blood into the brain. In particular, microglial cells become activated with LPS stimulus and produce ROS through the action of NADPH oxidase, TNFα, IL-1β, that impairs BBB function altering the expression of important molecules in the BBB integrity, such as ZO-1, claudin-5, occluding and P-gp. CD200-deficient mice showed an increased BBB permeability that possibly favors T cell entrance in the brain followed by an increased IFNγ expression that activates microglial cells and enhance the release of microglial activation markers including TNFα, IL-6, contributing to keeping the BBB injury.

Microglia are the resident immune cells in the CNS and perform an essential role in the immune response, while they are also an important component of the NVU (Spindler and Hsu, 2012). Microglia become activated under brain injury and immunological stimuli (Kreutzberg, 1996) and undergo several alterations from a “resting state” to an active state (Hanisch and Kettenmann, 2007; Kettenmann et al., 2011). This activation and consequent neuroinflammation are substantially involved in the progression of neurodegenerative diseases (McGeer and McGeer, 1995) and impairments of the BBB have been observed in this context (Dickstein et al., 2006; Zipser et al., 2007; Lassman et al., 2012; Figure 2).

Recent studies suggest that microglial activation may be related to BBB disruption. Sumi and collaborators evaluated the effects of lipopolysaccharide (LPS), a microglial activator, on BBB functions in an *in vitro* co-culture system using rat brain microvascular endothelial cells (RBEC) and microglia. Treatment with LPS on the outside of the insert (abluminal side) in both RBEC monolayer and RBEC/microglia co-culture showed no effect on transendothelial electrical resistance (TEER) in the RBEC monolayer. However, when the LPS treatment was performed on the RBEC/microglia co-culture, TEER was decreased and this was dependent on the number of microglial cells. Moreover, LPS had no effect on the permeability coefficient of sodium-fluorescein (Na-F) in RBEC monolayer, but in RBEC/microglia co-culture, LPS increased the Na-F permeability. The tight junctions and efflux transporters are the main machinery underlying the BBB function (Liebner et al., 2000; Rajasekaran et al., 2008). Immunostaining for the tight junction proteins zonula occludens-1 (ZO-1), claudin-5 and occludin exhibited a continuous distribution of these proteins along cell border in RBEC co-cultured with microglia, but when treated with LPS, this was changed to a expression pattern that was restored to a linear shape by adding DPI, a NADPH oxidase inhibitor. These results suggest that activated microglia probably produce reactive oxygen species (ROS), through NADPH oxidase, and impair BBB function (Sumi et al., 2010).

In fact, several neurodegenerative diseases include BBB dysfunction. For example, the disruption of BBB caused by oxidative stress seems to be an important step to the development of these disorders, since ROS have been shown to modulate BBB integrity by transient activation of PI3K/AKT pathway via RhoA leading to tight junction leakage (Schreibelt et al., 2007). In addition, the NADPH oxidase system of microglial cells seems to be the main producer of superoxide anion (O_2_^−^), causing BBB permeability (Block and Hong, 2005; Rojo et al., 2014). When microglial activation was inhibited with minocycline or superoxides produced by NADPH oxidase were blocked with apocynin, the BBB constituents were preserved *in vitro* whereas BBB disruption and hemorrhage were reduced *in vivo* (Yenari et al., 2006).

Furthermore, to clarify the involvement of tumor necrosis factor α (TNF-α) released from activated microglia on BBB integrity, an *in vitro* co-culture system with mouse brain capillary ECs (MBEC4) and microglia showed that the permeability of MBEC4 cells to Na-F was increased when microglial cells were activated by LPS and it was blocked by a neutralizing antibody against TNF-α, indicating that TNF-α contributes to BBB dysfunction (Nishioku et al., 2010). On the other hand, ICAM-1 plays an important role in cell-cell adhesive interactions and its expression in cerebral microvessels is low under normal conditions. However, ICAM-1 expression increases in the presence of proinflammatory mediators, such as TNF-α, interleukin (IL)-1β, IL-4 and interferon- γ (IFNγ). The correlation between induction of ICAM-1 expression and increased BBB permeability has been shown in acute inflammation. In an effort to understand by which pathways the peripheral inflammation affects BBB function and structure, Huber and collaborators investigated the effect of λ-carrageenan-induced inflammatory pain (CIP) on the ICAM-1 expression in the BBB. The induction of ICAM-1 was region-specific and directly correlated with an increase in microglial activation (Huber et al., 2006). Moreover, the decreased expression of CD200, which occurs with ageing or in the brain of Alzheimer’s Disease (AD) patients, is associated with microglial activation. CD200 is expressed on several cell types and its receptor, CD200R, is expressed on microglia. This interaction has been shown to be important in modulating inflammation and macrophage function. Denieffe and collaborators observed an increased expression of TNF-α and IFNγ in CD200 deficient mice, molecules known to promote classical activation of microglia. The increase of IFNγ levels was probably owing to the entrance of T cells and macrophages into the CNS facilitated by an increased BBB permeability, since IFNγ is normally not produced by resident cells in the brain (Denieffe et al., 2013).

Further evidence for the involvement of microglia on BBB opening was provided by the analysis of AD brain tissue, which showed overlap areas of microgliosis with fibrinogen immunoreactivity. Moreover, these representative patterns of staining were in apparent association with blood vessels. These findings incited *in vivo* experiments using intra-hippocampal injections of Aβ¬1–42 in rat brains, which caused time-dependent increase in expression of fibrinogen and microgliosis compared to the control. Moreover, IgG immunoreactivity was minimal in the control but significantly increased after Aβ¬1–42 injection. Inclusion of anti-Mac-1, a neutralizing monoclonal antibody that blocks the binding of fibrinogen to Mac-1 on microglia and inhibits microglial activation, was highly effective in decreasing IgG after Aβ¬1–42 injection. In these experiments, the permeability of BBB was determined by levels of IgG infiltration consistent with reduced microglial reactivity associated to an increased BBB preservation (Ryu and McLarnon, 2009). Therefore, microglial responses to Aβ¬1–42 can promote vascular changes that induce BBB breakdown plasma protein infiltration (Ryu and McLarnon, 2009).

Considering all the studies mentioned above, microglial activation impairs BBB function by the release of various molecules leading to a hyperpermeability condition associated with inflammation that is similar to what occurs in certain neurodegenerative disorders, including AD and Multiple Sclerosis (MS). We do not discard the entering and the action of bone marrow derived myeloid cells into the CNS in inflammatory conditions, but undoubtedly microglia certainly play an important role in these pathologies (Perry et al., 2010; Graeber et al., 2011).

An intact BBB is essential to control leukocyte trafficking either to immune response during brain infection, or after brain damage when microglial cells and macrophages must clear the debris (Shechter and Schwartz, 2013). On the other hand, the brain inflammatory response to antigen infiltration (virus, bacteria or fungus) triggered by activated microglia may result in aberrant expression of several chemokines, leading to alterations in BBB permeability (Buckner et al., 2006; Obermeier et al., 2013).

The microglial immune response to a brain infection is a complex event that involves several chemokines, particularly the monocyte chemoattractant protein-1 (MCP-1; Andjelkovic et al., 1999). MCP-1 is produced by several cell types including microglia, monocytes, macrophages, neurons, astrocytes and brain microvessel endothelial cells (BMECs; Andjelkovic et al., 1999; Andjelkovic and Pachter, 2000; Mahad and Ransohoff, 2003), and exerts biological functions by binding to its high affinity receptor CCR2, expressed by microglia, astrocytes and BMECs in the brain microenvironment (Banisadr et al., 2002; Ge et al., 2008). *In vivo* studies using MCP-1 knockout mice showed that this chemokine is important to maintenance of BBB integrity (Yao and Tsirka, 2011). On the other hand, the up-regulation of MCP-1 in the brain microenvironment during inflammatory response probably disrupts BBB integrity by redistribution of tight junction proteins, such as occludin and claudin-1, 5 and 11 and reorganization of the actin cytoskeleton in brain microvascular ECs (Stamatovic et al., 2003, 2005, 2006; Dimitrijevic et al., 2006).

Patients with bacterial/viral meningitis exhibit severe BBB impairment in the course of the disease resulting in brain edema, associated with high levels of TNF-α (Sharief et al., 1992; Glimåker et al., 1993; Leppert et al., 2000; Brivet et al., 2005; Ubenauf et al., 2007; Mook-Kanamori et al., 2011). During CNS infection by the human immunodeficiency virus (HIV), perivascular macrophages and microglia are the predominant cell types initially infected by HIV (Wiley et al., 1986). Infected monocytes are more responsive to MCP-1 than normal monocytes, which possibly increase their ability to cross the BBB through the accumulation of adhesion molecules, for instance E-selectin, in the interface between ECs from the BBB and monocytes, resulting in the infection of resident microglia and macrophages (Buckner et al., 2006; Eugenin et al., 2011). Cytokines are highly expressed in HIV-infected microglia/macrophage, leading in turn to an overexpression of IFNγ and TNF-α, inducing EC death pathway, and finally BBB dysfunction. Besides cytokine overexpression, viral proteins, mainly Tat, stimulate cyclooxygenase-2 (COX-2)—an enzyme produced in response to TNF-α during inflammation, associated with decrease of ZO-1 expression in the tight junctions, which leads to BBB rupture and various neurological disorders (Wang et al., 2008; Strazza et al., 2011).

In traumatic brain injury, the post-traumatic inflammatory response is related mainly to cytokine and metalloproteinase (MMP) expression in the lesion site, where IL-1β is associated with ZO-1 loss and tight junction redistribution (Bolton et al., 1998; Obermeier et al., 2013). The increased levels of MMPs, particularly MMP-2, 3 and 9, have also been observed, produced mainly by microglia (Truettner et al., 2005). These proteins disrupt the basal lamina proteins and degrade the tight junction complexes, resulting in BBB breakdown and severe neurological disorders after traumatic injury (Cunningham et al., 2005; Yang et al., 2007). Using a rat model of traumatic brain injury, Readnower and collaborators showed that microglial activation-derived oxygen free radicals as well as subsequent reaction products, hydrogen peroxide and nitric oxide (NO), have the potential to harm cells, contributing to oxidative damage, neurodegeneration and BBB impairment (Readnower et al., 2010; Briones et al., 2011; Xiong et al., 2012). One of the consequences of oxidative stress is the peroxidation of membrane polyunsaturated fatty acids, giving rise to active aldehydes significantly increasing ECs permeability (Chodobski et al., 2011). The toxicity of oxygen reactive species is observed in other cerebral disorders, such as ischemia (Massberg et al., 2002). During cerebral ischemia, there is platelet accumulation in microvessels, which triggers EC activation and increases ICAM-1 expression, enhancing the neutrophil infiltration in the brain parenchyma, contributing to cerebrovascular inflammation (Thornton et al., 2010).

### Epilepsy

According to the International League Against Epilepsy (ILAE) and the International Bureau for Epilepsy (IBE) a seizure is characterized by transient abnormal excessive or hypersynchronous neuronal activity in the CNS, which leads to specific signs and symptoms. The occurrence of more than one spontaneous seizure characterizes epilepsy, a brain disorder where there is enduring predisposition to generate such seizures (Fisher et al., 2005). Despite neurons being the “effector” cells in epilepsy, the importance of cells from the NVU, specially glial cells, in the pathogenesis of this disease becomes increasingly prominent.

Many works have acknowledged the role of neuroinflammation in the pathogenesis of seizures, but little is known about the mechanisms that start the inflammatory process in the CNS. On one hand, it is known that seizures can occur in many CNS diseases where inflammation contributes to its pathophysiology such as traumatic brain injury (Kadhim et al., 2008), ischemia (Denes et al., 2010; Downes and Crack, 2010), MS (Centonze et al., 2010) and AD (Eikelenboom et al., 2011). On the other hand, there is growing evidence that peripheral inflammation causes a “mirror” inflammatory response in the CNS, characterized by microglial and endothelial cytokine production and invasion of peripheral leukocytes (Quan et al., 1999; Qin et al., 2007; Riazi et al., 2008; Pyter et al., 2009), which can in turn reduce the threshold for seizure induction.

In the epileptic brain a key neuroinflammatory mediator is IL-1β, produced by microglia and astrocytes in great amounts (Ravizza et al., 2008a). Neurons and cells from the NVU (ECs, microglia and perivascular astrocytes) express IL-1R1 receptor and therefore, mediate the effects of this cytokine (Vezzani et al., 2008). In the context of seizures, astrocytes and neurons also produce other substances that modulate microglial function, such as TGF-β, ATP, HMGB1 (a chromatin associated nuclear protein) and fractalkine (CX_3_CL1; Verderio and Matteoli, 2001; Rodgers et al., 2009; Aronica et al., 2010, 2012; Dubé et al., 2010; Vezzani et al., 2011).

Microglial and astrocytic IL-1β act directly on the endothelium, altering BBB permeability and contributing to epileptogenesis. IL-1R1 activation on ECs increases BBB permeability through downregulation of tight junction proteins, mainly ZO-1, and upregulation of NO and MMPs (Ravizza et al., 2008a,b; Morin-Brureau et al., 2011; Librizzi et al., 2012). This BBB damage “from the inside” is associated with several downstream effects, all of which directly affect neuronal activity (Kofuji and Newman, 2004; Coulter and Eid, 2012). Endothelial cells activated by IL-1 β also have increased expression of adhesion molecules (ICAM-1, VCAM-1, E-Selectin and P-Selectin) that promote leukocyte adhesion, rolling and arrest in their luminal surface, releasing cytokines/proteases and damaging the BBB “from the outside”. This damage “from the outside” further alters BBB’s permeability to ions and proteins, contributing to sustain the mechanisms of hyperexcitability (Fujiwara and Kobayashi, 2005; Fabene et al., 2008; Kim et al., 2009).

The role of the other inflammatory mediators produced by microglia and astrocytes in the context of hyperexcitability is not as fully understood as IL-1β’s. Vascular endothelial growth factor, IL-6, TNF-α, CCL-2 and prostaglandins were also shown to be important for the development of seizures: IL-6 and CCL-2, for example, are important for chemoattraction of peripheral leukocytes that interact with ECs and contribute to BBB damage “from the outside” (Obermeier et al., 2013). Vascular endothelial growth factor, contributes to BBB damage by inducing downregulation of ZO-1, besides promoting microvascular proliferation (angiogenesis) via VEGFR2 on ECs (Ravizza et al., 2008a; Morin-Brureau et al., 2011; Librizzi et al., 2012). These studies further suggest the importance of the interaction between microglia and ECs in epilepsy.

In this context, the importance of microglial and astrocytic activation is corroborated by the control of epileptic syndromes resistant to conventional anti-epileptic drugs by anti-inflammatory or immunosuppressive treatments (Najjar et al., 2011). For example, intravenous immunoglobulin (IVIG) can reduce cytokine production and astrocyte activation and, therefore, suppresses seizures. Intravenous immunoglobulin increases the circulating levels of IL-1R antagonist (IL-1Ra), blocking IL-1β signaling (Crow et al., 2007; Mikati et al., 2010; Li et al., 2012). In addition, there are also ongoing clinical trials with VX-765, a selective inhibitor of caspase-1, the enzyme that cleaves the precursor form of IL-1β into the active peptide, and therefore reduces its production (Ravizza et al., 2008b; Maroso et al., 2011; Vezzani et al., 2011). Taking all the recent data into consideration, we can suggest that the cells of the NVU, specially glial cells, have an overwhelming importance in epileptogenesis. Excitation and inflammation, traditionally considered independent pathways, are now understood as overlapping and interconnected processes; as inflammation can promote excitability, and so can excitability promote inflammation. These current findings show the increasing importance of the inflammatory pathways activated by microglial cells in inducing BBB dysfunction, which contributes to epileptogenesis. Furthermore, this understanding is leading to the development of therapeutic strategies attempting to disrupt this self-perpetuating process of inflammation-hyperexcitability, aiming to improve the control of drug-resistant epilepsies.

### Stroke

The term *stroke* comprises a heterogeneous spectrum of conditions that have in common the interruption of blood supply to the brain parenchyma. This deficit in the blood flow leads to brain damage that is divided in two regions: the rapidly, severely injured necrotic core and the surrounding *penumbra* region (Liu et al., 2010; Ronaldson and Davis, 2012). The mechanisms of brain death in the ischemic core are mainly related to direct cellular damage due to oxygen and glucose deprivation, as a collapse of ion gradients and excitotoxicity (Adibhatla et al., 2006; Arai et al., 2011; Ronaldson and Davis, 2012). The importance of the crosstalk between neurons, ECs and glial cells (in particular microglia and astrocytes) becomes more prominent in the context of the *penumbra* region, a functionally impaired, but not dead area of the ischemic brain, which is pathophysiologically characterized by hypoxia/reoxygenation stress, BBB disruption, edema and active inflammation (Lo et al., 2005).

The re-establishment of the blood flow to the *penumbra* is responsible for most of the cellular damage observed in the ischemic stroke, associated with neuronal apoptosis, increase in cellular distress secondary to ROS production, and decreased concentrations of antioxidants (GSH). In this context, it is detachable the increased expression of hypoxia-inducible factor-1 (HIF-1) and nuclear factor-κB (NF-κB), in endothelial and glial cells, especially microglial cells. The pathways up-regulated by these transcription factors are involved in disruption of the BBB and neuroinflammation (Witt et al., 2005; Lochhead et al., 2010; Yang and Rosenberg, 2011). Besides that, activated microglia itself produce ROS and NO, further contributing to endothelial and neuronal damage, microglial activation, and perpetuation of stress.

One of the key components of the cell death cascades in stroke is neuroinflammation, where the main players are microglial cells and the peripheral leukocytes, especially neutrophils and monocytes/macrophages. After the ischemic insult, the recruitment of immune cells occurs in a biphasic dynamics. In the acute phase (within minutes), microglial cells are rapidly recruited to the injury site, due to the immediate release of cytokines and chemokines. These cells are activated, enhancing the release of ROS (through NADPH oxidase and MPO), cytokines (i.e., IL-1β, IL-6 and TNF-α) and chemokines (MCP-1, CXCL-1 and MIP-1α). At this time, ECs are also stimulated by these mediators, and the expression of adhesion molecules ICAM-1, P-Selectin and VCAM is upregulated, mainly by activation of the NF-κB pathway. These molecules are also upregulated in the circulating leukocytes, thus enhancing cell migration in the late phase (Kunsch and Medford, 1999; Jin et al., 2010; Enzmann et al., 2013; Obermeier et al., 2013). In this late phase, neutrophils and peripheral macrophages become important mediators of the neuroinflammation and propagation of the acute phase events, by sustaining the production of proinflammatory mediators and ROS, besides inducing and activating MMPs (Jin et al., 2010).

*In vivo* models helped clarifying the kinetics of the immune cells in ischemic stroke: Denes and colleagues used cell tracking techniques and MRI imaging to describe that, despite of BBB breakdown caused by transient middle cerebral artery occlusion (tMCAo), the infiltration of neutrophils was more prominent only in longer periods of MCAo, with extensive BBB damage. Also, very few infiltrating exogenous macrophages were observed over the first 72 h period and, instead, a profound increase in proliferating resident microglia cells was observed (Denes et al., 2007). However, microglia also remains as an important effector in the late phase, as Ekdahl and colleagues reported an increased number of activated microglial cells up to 16 weeks after two hour MCAO in rats (Ekdahl et al., 2009). Therefore, these works support the concept that microglia are a crucial mediator of the neuroinflammatory response in the CNS after ischemic injury, both in early and late phase stages.

Recently, the neuroinflammatory state was shown to activate the c-Jun N-terminal kinase (JNK) pathway not only in microglia but also in ECs, leading to microglial and endothelial activation with direct and microglial-induced BBB disruption, further cytokine production and perpetuation of the neuroinflammatory process (Tu et al., 2011; Wang et al., 2012).

BBB disruption is another key event in the pathogenesis of stroke, as it can worsen the clinical picture of the patients by the formation of intracerebral vasogenic edema and hemorrhagic transformation. As it occurs in neuroinflammation, BBB disruption has a biphasic dynamics. The early opening (12–48 h) of the BBB is mainly caused by oxidative stress, being ROS the most important mediator. They activate the latent MMP-2 min after the ischemic insult, and are produced by microglia and astrocytes through the HIF-1α pathway (Yang and Rosenberg, 2011; Obermeier et al., 2013). Another phenomenon observed in this early phase is the release of cytokine pools that were trapped by BBB ECM (“molecule trapping”), that activate glial and ECs and contribute to the recruitment of peripheral immune cells (Obermeier et al., 2013). In addition, ROS and NO can also reorganize the cytoskeleton of ECs and modulate tight junction proteins claudin-5 and occludin (Yamagata et al., 2004; Schreibelt et al., 2007) activate microglial cells; and up-regulate inflammatory mediators in the first 48 h. This microglial activation by ROS enhances the production of more ROS, intensifying the early response.

A delayed secondary opening of the BBB occurs after 48 to 72 h and results from the sustained inflammatory response in the brain parenchyma. In this phase, MMPs are important mediators: microglial ROS, especially superoxide radical O_2_^−^, increase vascular permeability also by stimulating MMP-2/MMP-3/MMP-9 production by microglia, astrocytes and ECs (Gottschall and Deb, 1996; Asahi et al., 2001; Rosenberg et al., 2001), contributing to hemorrhagic transformation and vasogenic edema. MMPs are further stimulated by NK-κB and HIF-1α and disrupt TJ proteins (as ZO-1, claudin-5 and occludin), rendering the BBB leaky, downstream effects which were shown to be reverted by blocking NOS (Yang and Rosenberg, 2011; Gu et al., 2012; Obermeier et al., 2013).

The activation of the previously stated pathways lead microglial cells to produce a variety of molecules, such as TNF-α, IL-1 β, IL-6, NO and insulin-like growth factor 1 (IGF-1) that interfere with BBB permeability. Apart from the effects of MMPs, microglial-induced late-phase BBB disruption is due to direct effects on ECs: IL-1β and TNF-α downregulate tight junction proteins expression in ECs (Yamagata et al., 2004; Jiao et al., 2011) and, together with IL-6, modulate the expression of adhesion molecules (ICAM-1 and VCAM; Hallenbeck, 2002). These actions not only help enhancing the influx of peripheral leukocytes, but also contribute to the formation of vasogenic edema by allowing the diffusion of sodium and water (Sandoval and Witt, 2008). These mediators also indirectly lead to BBB damage, by acting not on ECs, but on different components of the NVU: activating MMPs and acting on astrocytes and pericytes through ROS, NO and cytokines.

Blood-derived macrophages and microglia appear to have many functions in the dynamic process of CNS injury and repair, affecting BBB permeabilization, which in turn can aggravate stroke (Hallenbeck, 2002; Sandoval and Witt, 2008; Jiao et al., 2011). Preclinical and clinical studies corroborate this observation: minocycline, a member of the tetracycline antibiotic family, inhibits activation of microglia and brain infiltrating blood-derived macrophages and protects cultured neurons from excitotoxic insults by preventing microglial generation of glutamate, IL-1β and NO (Yrjänheikki et al., 1998, 1999; Tikka et al., 2001). These anti-inflammatory properties of minocycline are thought to act through inhibition of p38MAPK and MMP-9 (Tikka et al., 2001; Koistinaho et al., 2005). Inhibiting microglial activation may limit BBB disruption and reduce vasogenic edema in the context of ischemic stroke, reducing the volume of ischemic tissue and neuronal deficits, as well as preventing hemorrhagic transformation (Tikka et al., 2001; Yenari et al., 2006). So far, minocycline has been incorporated into two clinical trials involving ischemic stroke patients, demonstrating that minocycline administration (both alone and in combination with Fibrinolysis) improved neurological functional outcome following stroke (Fagan et al., 2011).

On the other hand, some groups advocate the concept that microglial cells are important for recovery from ischemic damage. Some i*n vitro* and *in vivo* studies have shown the role of IGF-1 in promoting neuroprotection and neuroregeneration (Li et al., 2010; Selvamani et al., 2012; Sohrabji and Williams, 2013; Bake et al., 2014). It has also been shown that the selective ablation of proliferating microglial cells in a transgenic mouse model exacerbates the extent of ischemic injury (Lalancette-Hébert et al., 2007). Besides that, Kitamura and colleagues demonstrated in a rat model that the intracerebroventricular injection of microglia protected the BBB and neurons against focal brain ischemia (Kitamura et al., 2004). All these data suggest that microglia has a role in the EC damage and BBB disruption in stroke, making these cells a possible target to minimize the ischemic damage in stroke patients.

### Neurodegenerative disorders

The BBB is seen as a protector of the brain homeostasis since it prevents the entry of several components from the blood into the brain parenchyma. In particular, during MS and AD, the mononuclear phagocytes from blood are also recruited via brain chemokines, which allows entry of cells from the bloodstream through the BBB (Britschgi and Wyss-Coray, 2007). Moreover, these inflammatory and neurodegenerative disorders cause an impairment of neural and synaptic functions due to the production of neurotoxic compounds (Zlokovic, 2008; Mizee and de Vries, 2013). MS and AD are quite distinct neurodegenerative diseases but both affect the CNS, leading to degeneration and consequently causing physical impairment and dementia, respectively.

Furthermore, several studies have shown that microglia can compromise BBB functions by the release of proinflammatory cytokines, such as TNF-α and IL-6, leading to BBB disruption (Britschgi and Wyss-Coray, 2007). Here, we will discuss the importance of microglia during the BBB breakdown in AD and MS.

### Alzheimer’s disease

Alzheimer’s disease is recognized as one of the most common causes of dementia that leads to impairment of memory, thinking and behavior in humans (Zlokovic, 2008). The major pathological features of this disease are the increased production and deposition of amyloid-β (Aβ) and intracellular accumulation of neurofibrillary tangle composed of hyperphosphorylated tau protein, besides synapse and neuronal loss (Takeda et al., 2014). Also, it is commonly accompanied by BBB dysfunction (Erickson and Banks, 2013; Lyros et al., 2014). In the initial stage of AD, there is loss of BBB homeostasis, leading to the production of proinflammatory cytokines and suppressors of the cerebral blood flow by ECs, which exacerbates synapse destruction, accumulation and activation of microglia. Also, in the late phase of AD, amyloid deposits are commonly observed in larger blood vessels and smaller cerebral capillaries (Zlokovic, 2004, 2011).

Crosstalk between systemic and central innate immune systems by the release of inflammatory mediators is observed in AD (Holmes, 2013). So, another role of BBB is the control of the entrance of T lymphocytes into the brain (Town et al., 2005). In AD patients, it was observed the presence of T cells in greater numbers than in non-AD patients (Monson et al., 2014). It was proved that the migration of T cells and immune cells into the brain occurs under inflammatory conditions. In this study it was shown that T cell migration into the brain through the BBB in AD depends on the TNF-α expressed by microglia, which induce the expression of MHC class I (MHC-I) on brain ECs (Yang et al., 2013).

The microglial cells and astrocytes are the resident brain cells responsible for the immune response in the brain, and it was observed in AD brains the presence of activated microglia and astrocytes around Aβ plaques, with the release of inflammatory cytokines, such as IL-1 and IL-6, TNF-α and transforming growth factor-β (TNF-β; Wyss-Coray, 2006; Zhou et al., 2012). In AD, these cells can promote the clearance of Aβ, however, if they are not able to do that, there is an accumulation of Aβ deposits leading to neuronal death (Rogers et al., 2002). It has already been shown that the elimination of Aβ from the brain and the consequent impairment of microglial activation decrease ROS, nitrogen compounds and also inflammatory cytokines produced by microglial cells, which are correlated with the activation of ECs (Dickstein et al., 2006). Several studies using animal models have further contributed with *in vivo* evidences of the importance of microglia during AD progression. In the study performed by El Khroury and collaborators using an AD transgenic model, a CCL2-deficient APP/PS1 mouse, which has a deficiency in the Ccr2 chemokine receptor and its ligand (CCL2) on microglia, they observed a regression in the disease (El Khoury et al., 2007; Kiyota et al., 2013). Moreover, high levels of Aβ are correlated with production of CCL2 by microglia, which leads to BBB impairment (Roberts et al., 2012).

On the other hand, it has been shown that LPS induces the production of TNF-α in the brain by microglial cells, which promotes BBB dysfunction and also causes neuronal cell death by phagocytosis (Tanaka et al., 2006; Nishioku et al., 2010; Smith et al., 2012; Neniskyte et al., 2014). Another cause of BBB breakdown is the accumulation of various molecules in the brain, such as thrombin, which is released by ECs, leading to activation of other components of the brain, like microglia and astrocytes. One study found that brain microvessels collected from AD patients produce high levels of thrombin, which directly affect neurons by the induction of cell death, and indirectly affect activation of microglia. Furthermore, it was shown that intracranial injection of thrombin activates microglia, leading to the production of NO and TNFα via JAK2-STAT3 signaling pathway, which induce BBB disruption. Thus, it appears that thrombin causes a disruption of BBB indirectly by activation of microglia (Huang et al., 2008a,b; Yin et al., 2010; Grammas, 2011).

Thus, regarding all evidences described above, we can hypothesize that microglia activation plays an important role during BBB disruption, which substantiates the need to study other factors correlated with inflammatory pathways, as potential targets of new strategies to reduce microglial-dependent inflammation in the brain of AD patients.

### Multiple sclerosis

Multiple sclerosis is a chronic, progressive and neuroinflammatory demyelinating disease of the CNS, which involves an energy deficit, tissue remodeling, microglial activation and loss of BBB integrity (Lassmann et al., 2012).

MS is often associated with the increase of BBB permeability, and consequently inflammatory response causing the formation of lesions and demyelization. Previous studies showed that systemic inflammation contributes to the disease progression and the activation of immune cells allows the infiltration of T and B cells into the CNS, promoting the release of chemokines that in turn stimulate the migration of more immune cells, leading to BBB disruption (Holman et al., 2011; Kooij et al., 2011; Larochelle et al., 2011; Willis, 2011). Moreover, during inflammation and demyelination in MS, the activation of microglial cells lead to the release of cytotoxic factors, such as NO and ROS, provoking myelin damage, release of proinflammatory cytokines (IFNγ, TNF-α and IL-1β), as well as chemokines (MCP-1), ultimately inducing BBB disruption (Mahad and Ransohoff, 2003). Activated microglia are known to express ROS-generating enzymes, such as NADPH oxidase, which are responsible for degeneration of oligodendrocytes and increased BBB permeability by downregulating the expression of VE-cadherin, occludin and claudin-5 proteins in microvascular ECs (Fischer et al., 2012; Rochfort et al., 2014).

Hereupon, there are two different types of inflammation in MS. The first consists in the initial response to the disease, involving the infiltration of CD4^+^ and CD8^+^ T-cells and robust microglial activation; the second occurs due to the myelin destruction, stimulating the secondary recruitment of T cells, B cells and macrophages (Lassmann et al., 2012). There are two well-established mouse models used to study MS, which have an ineffectual immune response: Theiler’s Murine Encephalomyelitis Virus-Induced Demyelinating Disease (TMEV-IDD; Rodriguez et al., 1987) and experimental autoimmune encephalomyelitis (EAE), where activation of microglia precedes infiltration of peripheral macrophages and occurs before the onset of the disease (Sriram and Steiner, 2005). *In vivo* imaging using two-photon laser scanning microscopy (2PLSM) on EAE animal showed dynamic interactions between BBB disruption and microglia activation. It was observed that microglial motility resulted from the leakage of the plasma protein fibrinogen before the onset of MS signs. In this event, fibrinogen induces the activation of microglia and consequently the release of ROS, inducing axonal damage (Davalos et al., 2012). Both in samples from patients with MS and in an animal model for MS, EAE, it was observed a high expression of MMPs. Microglia contributes to the inflammatory process through the production of MMPs, such as MMP−1, −2, −3, −9, and −19, which consequently leads to destabilization of the BBB permeability (Weaver et al., 2005; van Horssen et al., 2006).

Minocycline has been recognized as a microglia inhibitor (Li et al., 2005; Defaux et al., 2011; Kobayashi et al., 2013; Miron et al., 2013; Huang et al., 2014). Minocycline can reduce BBB breakdown by preventing microglial production of glutamate, MMPs, IL-1β and iNOS. The inhibition of microglial activation by minocycline favors the differentiation of oligodendrocyte precursors and immature oligodendrocytes. These cells are responsible for remyelination of neurons, an important event for improvement of MS condition (Li et al., 2005; Yenari et al., 2006; Defaux et al., 2011; Miron et al., 2013). Recently published data demonstrated that dipyridamole attenuates the expression of Toll-like receptor stimulation-dependent cytokines and chemokines in human microglia, reduces microglial activity in EAE mice, and consequently decreases IL-1β, TNF-α and IL-6 expression, which are responsible for increased BBB permeability (Sloka et al., 2013).

Taking into account the literature reviewed about AD and MS, we suggest that pharmacological modulation of microglial activation may control the impairment of BBB in these diseases.

## Concluding remarks

Over the last years, many studies have been performed aiming at understanding the role of BBB during ischemic injury, neurodegenerative disorders and infectious/inflammatory diseases. However, despite important advances made during the last decade, the mechanisms involved in the multifaceted interactions between the constituents of the NVU are not yet fully uncovered. In particular, how activation of microglial cells may play a key role in BBB disruption. When activated, microglia impair BBB function through the release of several inflammatory modulators leading to a hyperpermeability condition, which is associated with several brain disorders. In addition, the consequent infiltration of peripheral immune cells in turn affects microglia function. Microglial activation may be one of the earliest phenomena involved in the progression of these diseases. In this sense, the role of the inflammatory response triggered by the activated microglia seems to be essential to understand BBB dysfunction in CNS diseases. Therefore, efforts should be directed towards the development of new approaches focusing on microglia as a potential therapeutic target.

## Conflict of interest statement

The authors declare that the research was conducted in the absence of any commercial or financial relationships that could be construed as a potential conflict of interest.

## References

[B1] Adibhatla R. M. Hatcher J. F. Dempsey R. J. (2006). Lipids and lipidomics in brain injury and diseases. AAPS J. 8, E314–E321. 10.1208/aapsj080236 16796382 PMC3231558

[B2] Alliot F. Godin I. Pessac B. (1999). Microglia derive from progenitors, originating from the yolk sac and which proliferate in the brain. Brain Res. Dev. Brain Res. 117, 145–152. 10.1016/s0165-3806(99)00113-3 10567732

[B3] Andjelkovic A. V. Kerkovich D. Shanley J. Pulliam L. Pachter J. S. (1999). Expression of binding sites for beta chemokines on human astrocytes. Glia 28, 225–235. 10.1002/(sici)1098-1136(199912)28:3<225::aid-glia6>3.0.co;2-6 10559781

[B4] Andjelkovic A. V. Pachter J. S. (2000). Characterization of binding sites for chemokines MCP-1 and MIP-1alpha on human brain microvessels. J. Neurochem. 75, 1898–1906. 10.1046/j.1471-4159.2000.0751898.x 11032879

[B6] Arai K. Lok J. Guo S. Hayakawa K. Xing C. Lo E. H. (2011). Cellular mechanisms of neurovascular damage and repair after stroke. J. Child Neurol. 26, 1193–1198. 10.1177/0883073811408610 21628695 PMC3530192

[B7] Armulik A. Genové G. Mäe M. Nisancioglu M. H. Wallgard E. Niaudet C.. (2010). Pericytes regulate the blood-brain barrier. Nature 468, 557–561. 10.1038/nature09522 20944627

[B8] Aronica E. Fluiter K. Iyer A. Zurolo E. Vreijling J. Van Vliet E. A.. (2010). Expression pattern of miR-146a, an inflammation-associated microRNA, in experimental and human temporal lobe epilepsy. Eur. J. Neurosci. 31, 1100–1107. 10.1111/j.1460-9568.2010.07122.x 20214679

[B9] Aronica E. Ravizza T. Zurolo E. Vezzani A. (2012). Astrocyte immune responses in epilepsy. Glia 60, 1258–1268. 10.1002/glia.22312 22331574

[B10] Asahi M. Wang X. Mori T. Sumii T. Jung J. C. Moskowitz M. A.. (2001). Effects of matrix metalloproteinase-9 gene knock-out on the proteolysis of blood-brain barrier and white matter components after cerebral ischemia. J. Neurosci. 21, 7724–7732. 11567062 10.1523/JNEUROSCI.21-19-07724.2001 PMC6762894

[B11] Bake S. Selvamani A. Cherry J. Sohrabji F. (2014). Blood brain barrier and neuroinflammation are critical targets of IGF-1-mediated neuroprotection in stroke for middle-aged female rats. PLoS One 9:e91427. 10.1371/journal.pone.0091427 24618563 PMC3949985

[B12] Banisadr G. Quéraud-Lesaux F. Boutterin M. C. Pélaprat D. Zalc B. Rostène W.. (2002). Distribution, cellular localization and functional role of CCR2 chemokine receptors in adult rat brain. J. Neurochem. 81, 257–269. 10.1046/j.1471-4159.2002.00809.x 12064472

[B13] Block M. L. Hong J. S. (2005). Microglia and inflammation-mediated neurodegeneration: multiple triggers with a common mechanism. Prog. Neurobiol. 76, 77–98. 10.1016/j.pneurobio.2005.06.004 16081203

[B14] Bolton S. J. Anthony D. C. Perry V. H. (1998). Loss of the tight junction proteins occludin and zonula occludens-1 from cerebral vascular endothelium during neutrophil-induced blood-brain barrier breakdown in vivo. Neuroscience 86, 1245–1257. 10.1016/s0306-4522(98)00058-x 9697130

[B15] Briones T. L. Rogozinska M. Woods J. (2011). Modulation of ischemia-induced NMDAR1 activation by environmental enrichment decreases oxidative damage. J. Neurotrauma 28, 2485–2492. 10.1089/neu.2011.1842 21612313 PMC3235341

[B16] Britschgi M. Wyss-Coray T. (2007). Immune cells may fend off Alzheimer disease. Nat. Med. 13, 408–409. 10.1038/nm0407-408 17415372

[B17] Brivet F. G. Jacobs F. M. Mégarbane B. (2005). Cerebral output of cytokines in patients with pneumococcal meningitis. Crit. Care Med. 33, 2721–2722; author reply 2722–2723. 10.1097/01.ccm.0000187092.73841.82 16276227

[B18] Buckner C. M. Luers A. J. Calderon T. M. Eugenin E. A. Berman J. W. (2006). Neuroimmunity and the blood-brain barrier: molecular regulation of leukocyte transmigration and viral entry into the nervous system with a focus on neuroAIDS. J. Neuroimmune Pharmacol. 1, 160–181. 10.1007/s11481-006-9017-3 18040782 PMC4359614

[B19] Centonze D. Muzio L. Rossi S. Furlan R. Bernardi G. Martino G. (2010). The link between inflammation, synaptic transmission and neurodegeneration in multiple sclerosis. Cell Death Differ. 17, 1083–1091. 10.1038/cdd.2009.179 19927157

[B20] Checchin D. Sennlaub F. Levavasseur E. Leduc M. Chemtob S. (2006). Potential role of microglia in retinal blood vessel formation. Invest. Ophthalmol. Vis. Sci. 47, 3595–3602. 10.1167/iovs.05-1522 16877434

[B21] Chodobski A. Zink B. J. Szmydynger-Chodobska J. (2011). Blood-brain barrier pathophysiology in traumatic brain injury. Transl. Stroke Res. 2, 492–516. 10.1007/s12975-011-0125-x 22299022 PMC3268209

[B22] Coulter D. A. Eid T. (2012). Astrocytic regulation of glutamate homeostasis in epilepsy. Glia 60, 1215–1226. 10.1002/glia.22341 22592998 PMC3375386

[B23] Crow A. R. Song S. Semple J. W. Freedman J. Lazarus A. H. (2007). A role for IL-1 receptor antagonist or other cytokines in the acute therapeutic effects of IVIg? Blood 109, 155–158. 10.1182/blood-2006-05-023796 16954498

[B24] Cuadros M. A. Martin C. Coltey P. Almendros A. Navascués J. (1993). First appearance, distribution and origin of macrophages in the early development of the avian central nervous system. J. Comp. Neurol. 330, 113–129. 10.1002/cne.903300110 8468399

[B25] Cunningham A. S. Salvador R. Coles J. P. Chatfield D. A. Bradley P. G. Johnston A. J.. (2005). Physiological thresholds for irreversible tissue damage in contusional regions following traumatic brain injury. Brain 128, 1931–1942. 10.1093/brain/awh536 15888537

[B26] Daneman R. Zhou L. Kebede A. A. Barres B. A. (2010). Pericytes are required for blood-brain barrier integrity during embryogenesis. Nature 468, 562–566. 10.1038/nature09513 20944625 PMC3241506

[B27] Davalos D. Ryu J. K. Merlini M. Baeten K. M. Le Moan N. Petersen M. A.. (2012). Fibrinogen-induced perivascular microglial clustering is required for the development of axonal damage in neuroinflammation. Nat. Commun. 3:1227. 10.1038/ncomms2230 23187627 PMC3514498

[B28] Defaux A. Zurich M. G. Honegger P. Monnet-Tschudi F. (2011). Minocycline promotes remyelination in aggregating rat brain cell cultures after interferon-*γ* plus lipopolysaccharide-induced demyelination. Neuroscience 187, 84–92. 10.1016/j.neuroscience.2011.04.053 21549181

[B29] Denes A. Thornton P. Rothwell N. J. Allan S. M. (2010). Inflammation and brain injury: acute cerebral ischaemia, peripheral and central inflammation. Brain Behav. Immun. 24, 708–723. 10.1016/j.bbi.2009.09.010 19770034

[B30] Denes A. Vidyasagar R. Feng J. Narvainen J. McColl B. W. Kauppinen R. A.. (2007). Proliferating resident microglia after focal cerebral ischaemia in mice. J. Cereb. Blood Flow Metab. 27, 1941–1953. 10.1038/sj.jcbfm.9600495 17440490

[B31] Denieffe S. Kelly R. J. McDonald C. Lyons A. Lynch M. A. (2013). Classical activation of microglia in CD200-deficient mice is a consequence of blood brain barrier permeability and infiltration of peripheral cells. Brain Behav. Immun. 34, 86–97. 10.1016/j.bbi.2013.07.174 23916893

[B32] Dickstein D. L. Biron K. E. Ujiie M. Pfeifer C. G. Jeffries A. R. Jefferies W. A. (2006). Abeta peptide immunization restores blood-brain barrier integrity in Alzheimer disease. FASEB J. 20, 426–433. 10.1096/fj.05-3956com 16507760

[B33] Dimitrijevic O. B. Stamatovic S. M. Keep R. F. Andjelkovic A. V. (2006). Effects of the chemokine CCL2 on blood-brain barrier permeability during ischemia-reperfusion injury. J. Cereb. Blood Flow Metab. 26, 797–810. 10.1038/sj.jcbfm.9600229 16192992

[B34] Downes C. E. Crack P. J. (2010). Neural injury following stroke: are toll-like receptors the link between the immune system and the CNS? Br. J. Pharmacol. 160, 1872–1888. 10.1111/j.1476-5381.2010.00864.x 20649586 PMC2958633

[B35] Dubé C. M. Ravizza T. Hamamura M. Zha Q. Keebaugh A. Fok K.. (2010). Epileptogenesis provoked by prolonged experimental febrile seizures: mechanisms and biomarkers. J. Neurosci. 30, 7484–7494. 10.1523/JNEUROSCI.0551-10.2010 20519523 PMC2906240

[B36] Eikelenboom P. Veerhuis R. van Exel E. Hoozemans J. J. Rozemuller A. J. van Gool W. A. (2011). The early involvement of the innate immunity in the pathogenesis of late-onset Alzheimer’s disease: neuropathological, epidemiological and genetic evidence. Curr. Alzheimer Res. 8, 142–150. 10.2174/156720511795256080 21345167

[B37] Ekdahl C. T. Kokaia Z. Lindvall O. (2009). Brain inflammation and adult neurogenesis: the dual role of microglia. Neuroscience 158, 1021–1029. 10.1016/j.neuroscience.2008.06.052 18662748

[B38] El Khoury J. Toft M. Hickman S. E. Means T. K. Terada K. Geula C.. (2007). Ccr2 deficiency impairs microglial accumulation and accelerates progression of Alzheimer-like disease. Nat. Med. 13, 432–438. 10.1038/nm1555 17351623

[B39] Enzmann G. Mysiorek C. Gorina R. Cheng Y. J. Ghavampour S. Hannocks M. J.. (2013). The neurovascular unit as a selective barrier to polymorphonuclear granulocyte (PMN) infiltration into the brain after ischemic injury. Acta Neuropathol. 125, 395–412. 10.1007/s00401-012-1076-3 23269317 PMC3578720

[B40] Erickson M. A. Banks W. A. (2013). Blood-brain barrier dysfunction as a cause and consequence of Alzheimer’s disease. J. Cereb. Blood Flow Metab. 33, 1500–1513. 10.1038/jcbfm.2013.135 23921899 PMC3790938

[B41] Espinosa-Heidmann D. G. Suner I. J. Hernandez E. P. Monroy D. Csaky K. G. Cousins S. W. (2003). Macrophage depletion diminishes lesion size and severity in experimental choroidal neovascularization. Invest. Ophthalmol. Vis. Sci. 44, 3586–3592. 10.1167/iovs.03-0038 12882811

[B42] Eugenin E. A. Clements J. E. Zink M. C. Berman J. W. (2011). Human immunodeficiency virus infection of human astrocytes disrupts blood-brain barrier integrity by a gap junction-dependent mechanism. J. Neurosci. 31, 9456–9465. 10.1523/JNEUROSCI.1460-11.2011 21715610 PMC3132881

[B43] Fabene P. F. Navarro Mora G. Martinello M. Rossi B. Merigo F. Ottoboni L.. (2008). A role for leukocyte-endothelial adhesion mechanisms in epilepsy. Nat. Med. 14, 1377–1383. 10.1038/nm.1878 19029985 PMC2710311

[B44] Fagan S. C. Cronic L. E. Hess D. C. (2011). Minocycline development for acute ischemic stroke. Transl. Stroke Res. 2, 202–208. 10.1007/s12975-011-0072-6 21909339 PMC3169090

[B45] Fantin A. Vieira J. M. Gestri G. Denti L. Schwarz Q. Prykhozhij S.. (2010). Tissue macrophages act as cellular chaperones for vascular anastomosis downstream of VEGF-mediated endothelial tip cell induction. Blood 116, 829–840. 10.1182/blood-2009-12-257832 20404134 PMC2938310

[B46] Fischer M. T. Sharma R. Lim J. L. Haider L. Frischer J. M. Drexhage J.. (2012). NADPH oxidase expression in active multiple sclerosis lesions in relation to oxidative tissue damage and mitochondrial injury. Brain 135, 886–899. 10.1093/brain/aws012 22366799 PMC3286337

[B47] Fisher R. S. van Emde Boas W. Blume W. Elger C. Genton P. Lee P.. (2005). Epileptic seizures and epilepsy: definitions proposed by the International League Against Epilepsy (ILAE) and the International Bureau for Epilepsy (IBE). Epilepsia 46, 470–472. 10.1111/j.0013-9580.2005.66104.x 15816939

[B48] Fujiwara N. Kobayashi K. (2005). Macrophages in inflammation. Curr. Drug Targets Inflamm. Allergy 4, 281–286. 10.2174/1568010054022024 16101534

[B49] Ge S. Song L. Serwanski D. R. Kuziel W. A. Pachter J. S. (2008). Transcellular transport of CCL2 across brain microvascular endothelial cells. J. Neurochem. 104, 1219–1232. 10.1111/j.1471-4159.2007.05056.x 18289346

[B50] Gerhardt H. Golding M. Fruttiger M. Ruhrberg C. Lundkvist A. Abramsson A.. (2003). VEGF guides angiogenic sprouting utilizing endothelial tip cell filopodia. J. Cell Biol. 161, 1163–1177. 10.1083/jcb.200302047 12810700 PMC2172999

[B51] Ginhoux F. Greter M. Leboeuf M. Nandi S. See P. Gokhan S. (2010). Fate mapping analysis reveals that adult microglia derive from primitive macrophages. Science 330, 841–845. 10.1126/science.1194637 20966214 PMC3719181

[B52] Glimåker M. Kragsbjerg P. Forsgren M. Olcén P. (1993). Tumor necrosis factor-alpha (TNF alpha) in cerebrospinal fluid from patients with meningitis of different etiologies: high levels of TNF alpha indicate bacterial meningitis. J. Infect. Dis. 167, 882–889. 10.1093/infdis/167.4.882 8450254

[B53] Gottschall P. E. Deb S. (1996). Regulation of matrix metalloproteinase expressions in astrocytes, microglia and neurons. Neuroimmunomodulation 3, 69–75. 10.1159/000097229 8945720

[B54] Graeber M. B. Li W. Rodriguez M. L. (2011). Role of microglia in CNS inflammation. FEBS Lett. 585, 3798–3805. 10.1016/j.febslet.2011.08.033 21889505

[B55] Grammas P. (2011). Neurovascular dysfunction, inflammation and endothelial activation: implications for the pathogenesis of Alzheimer’s disease. J. Neuroinflammation 8:26. 10.1186/1742-2094-8-26 21439035 PMC3072921

[B56] Gu Y. Zheng G. Xu M. Li Y. Chen X. Zhu W.. (2012). Caveolin-1 regulates nitric oxide-mediated matrix metalloproteinases activity and blood-brain barrier permeability in focal cerebral ischemia and reperfusion injury. J. Neurochem. 120, 147–156. 10.1111/j.1471-4159.2011.07542.x 22007835

[B57] Hallenbeck J. M. (2002). The many faces of tumor necrosis factor in stroke. Nat. Med. 8, 1363–1368. 10.1038/nm1202-1363 12457181

[B58] Hanisch U. K. Kettenmann H. (2007). Microglia: active sensor and versatile effector cells in the normal and pathologic brain. Nat. Neurosci. 10, 1387–1394. 10.1038/nn1997 17965659

[B60] Holman D. W. Klein R. S. Ransohoff R. M. (2011). The blood-brain barrier, chemokines and multiple sclerosis. Biochim. Biophys. Acta 1812, 220–230. 10.1016/j.bbadis.2010.07.019 20692338 PMC3005102

[B61] Holmes C. (2013). Review: systemic inflammation and Alzheimer’s disease. Neuropathol. Appl. Neurobiol. 39, 51–68. 10.1111/j.1365-2990.2012.01307.x 23046210

[B62] Huang C. Y. Chen Y. L. Li A. H. Lu J. C. Wang H. L. (2014). Minocycline, a microglial inhibitor, blocks spinal CCL2-induced heat hyperalgesia and augmentation of glutamatergic transmission in substantia gelatinosa neurons. J. Neuroinflammation 11:7. 10.1186/1742-2094-11-7 24405660 PMC3896825

[B63] Huang C. F. Li G. Ma R. Sun S. G. Chen J. G. (2008b). Thrombin-induced microglial activation contributes to the degeneration of nigral dopaminergic neurons in vivo. Neurosci. Bull. 24, 66–72. 10.1007/s12264-008-0066-x 18369384 PMC5552513

[B64] Huang C. Ma R. Sun S. Wei G. Fang Y. Liu R.. (2008a). JAK2-STAT3 signaling pathway mediates thrombin-induced proinflammatory actions of microglia in vitro. J. Neuroimmunol. 204, 118–125. 10.1016/j.jneuroim.2008.07.004 18710787

[B65] Huber J. D. Campos C. R. Mark K. S. Davis T. P. (2006). Alterations in blood-brain barrier ICAM-1 expression and brain microglial activation after lambda-carrageenan-induced inflammatory pain. Am. J. Physiol. Heart Circ. Physiol. 290, H732–H740. 10.1152/ajpheart.00747.2005 16199477 PMC3915803

[B66] Iadecola C. (2004). Neurovascular regulation in the normal brain and in Alzheimer’s disease. Nat. Rev. Neurosci. 5, 347–360. 10.1038/nrn1387 15100718

[B67] Janzer R. C. Raff M. C. (1987). Astrocytes induce blood-brain barrier properties in endothelial cells. Nature 325, 253–257. 10.1038/325253a0 3543687

[B68] Jiao H. Wang Z. Liu Y. Wang P. Xue Y. (2011). Specific role of tight junction proteins claudin-5, occludin and ZO-1 of the blood-brain barrier in a focal cerebral ischemic insult. J. Mol. Neurosci. 44, 130–139. 10.1007/s12031-011-9496-4 21318404

[B69] Jin R. Yang G. Li G. (2010). Inflammatory mechanisms in ischemic stroke: role of inflammatory cells. J. Leukoc. Biol. 87, 779–789. 10.1189/jlb.1109766 20130219 PMC2858674

[B70] Kadhim H. J. Duchateau J. Sébire G. (2008). Cytokines and brain injury: invited review. J. Intensive Care Med. 23, 236–249. 10.1177/0885066608318458 18504260

[B71] Kettenmann H. Hanisch U. K. Noda M. Verkhratsky A. (2011). Physiology of microglia. Physiol. Rev. 91, 461–553. 10.1152/physrev.00011.2010 21527731

[B72] Kim J. V. Kang S. S. Dustin M. L. McGavern D. B. (2009). Myelomonocytic cell recruitment causes fatal CNS vascular injury during acute viral meningitis. Nature 457, 191–195. 10.1038/nature07591 19011611 PMC2702264

[B73] Kitamura Y. Takata K. Inden M. Tsuchiya D. Yanagisawa D. Nakata J.. (2004). Intracerebroventricular injection of microglia protects against focal brain ischemia. J. Pharmacol. Sci. 94, 203–206. 10.1254/jphs.94.203 14978360

[B74] Kiyota T. Gendelman H. E. Weir R. A. Higgins E. E. Zhang G. Jain M. (2013). CCL2 affects *β*-amyloidosis and progressive neurocognitive dysfunction in a mouse model of Alzheimer’s disease. Neurobiol. Aging 34, 1060–1068. 10.1016/j.neurobiolaging.2012.08.009 23040664 PMC4011558

[B75] Kobayashi K. Imagama S. Ohgomori T. Hirano K. Uchimura K. Sakamoto K.. (2013). Minocycline selectively inhibits M1 polarization of microglia. Cell Death Dis. 4:e525. 10.1038/cddis.2013.54 23470532 PMC3613832

[B76] Kofuji P. Newman E. A. (2004). Potassium buffering in the central nervous system. Neuroscience 129, 1045–1056. 10.1016/j.neuroscience.2004.06.008 15561419 PMC2322935

[B77] Koistinaho M. Malm T. M. Kettunen M. I. Goldsteins G. Starckx S. Kauppinen R. A.. (2005). Minocycline protects against permanent cerebral ischemia in wild type but not in matrix metalloprotease-9-deficient mice. J. Cereb. Blood Flow Metab. 25, 460–467. 10.1038/sj.jcbfm.9600040 15674236

[B78] Kooij G. Mizee M. R. van Horssen J. Reijerkerk A. Witte M. E. Drexhage J. A.. (2011). Adenosine triphosphate-binding cassette transporters mediate chemokine (C-C motif) ligand 2 secretion from reactive astrocytes: relevance to multiple sclerosis pathogenesis. Brain 134, 555–570. 10.1093/brain/awq330 21183485

[B79] Kreutzberg G. W. (1996). Microglia: a sensor for pathological events in the CNS. Trends Neurosci. 19, 312–318. 10.1016/0166-2236(96)10049-7 8843599

[B80] Kunsch C. Medford R. M. (1999). Oxidative stress as a regulator of gene expression in the vasculature. Circ. Res. 85, 753–766. 10.1161/01.res.85.8.753 10521248

[B81] Lalancette-Hébert M. Gowing G. Simard A. Weng Y. C. Kriz J. (2007). Selective ablation of proliferating microglial cells exacerbates ischemic injury in the brain. J. Neurosci. 27, 2596–2605. 10.1523/jneurosci.5360-06.2007 17344397 PMC6672496

[B82] Larochelle C. Alvarez J. I. Prat A. (2011). How do immune cells overcome the blood-brain barrier in multiple sclerosis? FEBS Lett. 585, 3770–3780. 10.1016/j.febslet.2011.04.066 21550344

[B83] Lassman M. E. McLaughlin T. M. Somers E. P. Stefanni A. C. Chen Z. Murphy B. A.. (2012). A rapid method for cross-species quantitation of apolipoproteins A1, B48 and B100 in plasma by ultra-performance liquid chromatography/tandem mass spectrometry. Rapid Commun. Mass Spectrom. 26, 101–108. 10.1002/rcm.5296 22173797

[B84] Lassmann H. van Horssen J. Mahad D. (2012). Progressive multiple sclerosis: pathology and pathogenesis. Nat. Rev. Neurol. 8, 647–656. 10.1038/nrneurol.2012.168 23007702

[B85] Lenard A. Ellertsdottir E. Herwig L. Krudewig A. Sauteur L. Belting H. G.. (2013). In vivo analysis reveals a highly stereotypic morphogenetic pathway of vascular anastomosis. Dev. Cell 25, 492–506. 10.1016/j.devcel.2013.05.010 23763948

[B86] Leppert D. Leib S. L. Grygar C. Miller K. M. Schaad U. B. Holländer G. A. (2000). Matrix metalloproteinase (MMP)-8 and MMP-9 in cerebrospinal fluid during bacterial meningitis: association with blood-brain barrier damage and neurological sequelae. Clin. Infect. Dis. 31, 80–84. 10.1086/313922 10913401

[B87] Li D. Li P. He Z. Cen D. Meng Z. Liang L.. (2012). Human intravenous immunoglobulins suppress seizure activities and inhibit the activation of GFAP-positive astrocytes in the hippocampus of picrotoxin-kindled rats. Int. J. Neurosci. 122, 200–208. 10.3109/00207454.2011.639470 22082354

[B88] Li S. Overman J. J. Katsman D. Kozlov S. V. Donnelly C. J. Twiss J. L.. (2010). An age-related sprouting transcriptome provides molecular control of axonal sprouting after stroke. Nat. Neurosci. 13, 1496–1504. 10.1038/nn.2674 21057507 PMC3059556

[B89] Li W. W. Setzu A. Zhao C. Franklin R. J. (2005). Minocycline-mediated inhibition of microglia activation impairs oligodendrocyte progenitor cell responses and remyelination in a non-immune model of demyelination. J. Neuroimmunol. 158, 58–66. 10.1016/j.jneuroim.2004.08.011 15589038

[B90] Librizzi L. Noè F. Vezzani A. de Curtis M. Ravizza T. (2012). Seizure-induced brain-borne inflammation sustains seizure recurrence and blood-brain barrier damage. Ann. Neurol. 72, 82–90. 10.1002/ana.23567 22829270

[B91] Liebner S. Kniesel U. Kalbacher H. Wolburg H. (2000). Correlation of tight junction morphology with the expression of tight junction proteins in blood-brain barrier endothelial cells. Eur J. Cell Biol. 79, 707–717. 10.1078/0171-9335-00101 11089919

[B92] Lindahl P. Johansson B. R. Levéen P. Betsholtz C. (1997). Pericyte loss and microaneurysm formation in PDGF-B-deficient mice. Science 277, 242–245. 10.1126/science.277.5323.242 9211853

[B93] Liu S. Levine S. R. Winn H. R. (2010). Targeting ischemic penumbra: part I—from pathophysiology to therapeutic strategy. J. Exp. Stroke Transl. Med. 3, 47–55. 10.6030/1939-067x-3.1.47 20607107 PMC2896002

[B94] Lo E. H. Moskowitz M. A. Jacobs T. P. (2005). Exciting, radical, suicidal: how brain cells die after stroke. Stroke 36, 189–192. 10.1161/01.str.0000153069.96296.fd 15637315

[B95] Lochhead J. J. McCaffrey G. Quigley C. E. Finch J. DeMarco K. M. Nametz N.. (2010). Oxidative stress increases blood-brain barrier permeability and induces alterations in occludin during hypoxia-reoxygenation. J. Cereb. Blood Flow Metab. 30, 1625–1636. 10.1038/jcbfm.2010.29 20234382 PMC2949263

[B96] Lyros E. Bakogiannis C. Liu Y. Fassbender K. (2014). Molecular links between endothelial dysfunction and neurodegeneration in Alzheimer’s disease. Curr. Alzheimer Res. 11, 18–26. 10.2174/1567205010666131119235254 24251393

[B97] Mahad D. J. Ransohoff R. M. (2003). The role of MCP-1 (CCL2) and CCR2 in multiple sclerosis and experimental autoimmune encephalomyelitis (EAE). Semin. Immunol. 15, 23–32. 10.1016/s1044-5323(02)00125-2 12495638

[B98] Maroso M. Balosso S. Ravizza T. Iori V. Wright C. I. French J.. (2011). Interleukin-1*β* biosynthesis inhibition reduces acute seizures and drug resistant chronic epileptic activity in mice. Neurotherapeutics 8, 304–315. 10.1007/s13311-011-0039-z 21431948 PMC3101825

[B99] Massberg S. Brand K. Grüner S. Page S. Müller E. Müller I.. (2002). A critical role of platelet adhesion in the initiation of atherosclerotic lesion formation. J. Exp. Med. 196, 887–896. 10.1084/jem.20012044 12370251 PMC2194025

[B100] Mathiisen T. M. Lehre K. P. Danbolt N. C. Ottersen O. P. (2010). The perivascular astroglial sheath provides a complete covering of the brain microvessels: an electron microscopic 3D reconstruction. Glia 58, 1094–1103. 10.1002/glia.20990 20468051

[B102] McGeer P. L. McGeer E. G. (1995). The inflammatory response system of brain: implications for therapy of Alzheimer and other neurodegenerative diseases. Brain Res. Brain Res. Rev. 21, 195–218. 10.1016/0165-0173(95)00011-9 8866675

[B103] Mikati M. A. Kurdi R. El-Khoury Z. Rahi A. Raad W. (2010). Intravenous immunoglobulin therapy in intractable childhood epilepsy: open-label study and review of the literature. Epilepsy Behav. 17, 90–94. 10.1016/j.yebeh.2009.10.020 20004620

[B104] Miron V. E. Boyd A. Zhao J. W. Yuen T. J. Ruckh J. M. Shadrach J. L.. (2013). M2 microglia and macrophages drive oligodendrocyte differentiation during CNS remyelination. Nat. Neurosci. 16, 1211–1218. 10.1038/nn.3469 23872599 PMC3977045

[B105] Mizee M. R. de Vries H. E. (2013). Blood-brain barrier regulation: environmental cues controlling the onset of barrier properties. Tissue Barriers 1:e26882. 10.4161/tisb.26882 24868496 PMC3943847

[B106] Monson N. L. Ireland S. J. Ligocki A. J. Chen D. Rounds W. H. Li M.. (2014). Elevated CNS inflammation in patients with preclinical Alzheimer’s disease. J. Cereb. Blood Flow Metab. 34, 30–33. 10.1038/jcbfm.2013.183 24149932 PMC3887357

[B107] Mook-Kanamori B. B. Geldhoff M. van der Poll T. van de Beek D. (2011). Pathogenesis and pathophysiology of pneumococcal meningitis. Clin. Microbiol. Rev. 24, 557–591. 10.1128/CMR.00008-11 21734248 PMC3131058

[B108] Morin-Brureau M. Lebrun A. Rousset M. C. Fagni L. Bockaert J. de Bock F.. (2011). Epileptiform activity induces vascular remodeling and zonula occludens 1 downregulation in organotypic hippocampal cultures: role of VEGF signaling pathways. J. Neurosci. 31, 10677–10688. 10.1523/JNEUROSCI.5692-10.2011 21775611 PMC6622643

[B110] Najjar S. Pearlman D. Miller D. C. Devinsky O. (2011). Refractory epilepsy associated with microglial activation. Neurologist 17, 249–254. 10.1097/NRL.0b013e31822aad04 21881466

[B111] Nayak D. Roth T. L. McGavern D. B. (2014). Microglia development and function. Annu. Rev. Immunol. 32, 367–402. 10.1146/annurev-immunol-032713-120240 24471431 PMC5001846

[B112] Neniskyte U. Vilalta A. Brown G. C. (2014). Tumour necrosis factor alpha-induced neuronal loss is mediated by microglial phagocytosis. FEBS Lett. 588, 2952–2956. 10.1016/j.febslet.2014.05.046 24911209 PMC4158418

[B113] Nishioku T. Matsumoto J. Dohgu S. Sumi N. Miyao K. Takata F.. (2010). Tumor necrosis factor-alpha mediates the blood-brain barrier dysfunction induced by activated microglia in mouse brain microvascular endothelial cells. J. Pharmacol. Sci. 112, 251–254. 10.1254/jphs.09292sc 20118615

[B114] Obermeier B. Daneman R. Ransohoff R. M. (2013). Development, maintenance and disruption of the blood-brain barrier. Nat. Med. 19, 1584–1596. 10.1038/nm.3407 24309662 PMC4080800

[B115] Perry V. H. Nicoll J. A. Holmes C. (2010). Microglia in neurodegenerative disease. Nat. Rev. Neurol. 6, 193–201. 10.1038/nrneurol.2010.17 20234358

[B116] Pyter L. M. Pineros V. Galang J. A. McClintock M. K. Prendergast B. J. (2009). Peripheral tumors induce depressive-like behaviors and cytokine production and alter hypothalamic-pituitary-adrenal axis regulation. Proc. Natl. Acad. Sci. U S A 106, 9069–9074. 10.1073/pnas.0811949106 19451634 PMC2689998

[B117] Qin L. Wu X. Block M. L. Liu Y. Breese G. R. Hong J. S.. (2007). Systemic LPS causes chronic neuroinflammation and progressive neurodegeneration. Glia 55, 453–462. 10.1002/glia.20467 17203472 PMC2871685

[B118] Quan N. Stern E. L. Whiteside M. B. Herkenham M. (1999). Induction of pro-inflammatory cytokine mRNAs in the brain after peripheral injection of subseptic doses of lipopolysaccharide in the rat. J. Neuroimmunol. 93, 72–80. 10.1016/s0165-5728(98)00193-3 10378870

[B119] Rajasekaran S. A. Beyenbach K. W. Rajasekaran A. K. (2008). Interactions of tight junctions with membrane channels and transporters. Biochim. Biophys. Acta 1778, 757–769. 10.1016/j.bbamem.2007.11.007 18086552

[B120] Ransohoff R. M. Engelhardt B. (2012). The anatomical and cellular basis of immune surveillance in the central nervous system. Nat. Rev. Immunol. 12, 623–635. 10.1038/nri3265 22903150

[B121] Ravizza T. Gagliardi B. Noé F. Boer K. Aronica E. Vezzani A. (2008a). Innate and adaptive immunity during epileptogenesis and spontaneous seizures: evidence from experimental models and human temporal lobe epilepsy. Neurobiol. Dis. 29, 142–160. 10.1016/j.nbd.2007.08.012 17931873

[B122] Ravizza T. Noe F. Zardoni D. Vaghi V. Sifringer M. Vezzani A. (2008b). Interleukin converting enzyme inhibition impairs kindling epileptogenesis in rats by blocking astrocytic IL-1beta production. Neurobiol. Dis. 31, 327–333. 10.1016/j.nbd.2008.05.007 18632279

[B123] Readnower R. D. Chavko M. Adeeb S. Conroy M. D. Pauly J. R. McCarron R. M.. (2010). Increase in blood-brain barrier permeability, oxidative stress and activated microglia in a rat model of blast-induced traumatic brain injury. J. Neurosci. Res. 88, 3530–3539. 10.1002/jnr.22510 20882564 PMC2965798

[B124] Riazi K. Galic M. A. Kuzmiski J. B. Ho W. Sharkey K. A. Pittman Q. J. (2008). Microglial activation and TNFalpha production mediate altered CNS excitability following peripheral inflammation. Proc. Natl. Acad. Sci. U S A 105, 17151–17156. 10.1073/pnas.0806682105 18955701 PMC2579393

[B125] Roberts T. K. Eugenin E. A. Lopez L. Romero I. A. Weksler B. B. Couraud P. O.. (2012). CCL2 disrupts the adherens junction: implications for neuroinflammation. Lab. Invest. 92, 1213–1233. 10.1038/labinvest.2012.80 22641100 PMC3409314

[B126] Rochfort K. D. Collins L. E. Murphy R. P. Cummins P. M. (2014). Downregulation of blood-brain barrier phenotype by proinflammatory cytokines involves NADPH oxidase-dependent ROS generation: consequences for interendothelial adherens and tight junctions. PLoS One 9:e101815. 10.1371/journal.pone.0101815 24992685 PMC4081725

[B127] Rodgers K. M. Hutchinson M. R. Northcutt A. Maier S. F. Watkins L. R. Barth D. S. (2009). The cortical innate immune response increases local neuronal excitability leading to seizures. Brain 132, 2478–2486. 10.1093/brain/awp177 19567702 PMC2732268

[B128] Rodriguez M. Oleszak E. Leibowitz J. (1987). Theiler’s murine encephalomyelitis: a model of demyelination and persistence of virus. Crit. Rev. Immunol. 7, 325–365. 2827957

[B129] Rogers J. Strohmeyer R. Kovelowski C. J. Li R. (2002). Microglia and inflammatory mechanisms in the clearance of amyloid beta peptide. Glia 40, 260–269. 10.1002/glia.10153 12379913

[B130] Rojo A. I. McBean G. Cindric M. Egea J. López M. G. Rada P.. (2014). Redox control of microglial function: molecular mechanisms and functional significance. Antioxid. Redox Signal. 21, 1766–1801. 10.1089/ars.2013.5745 24597893 PMC4186766

[B131] Ronaldson P. T. Davis T. P. (2012). Blood-brain barrier integrity and glial support: mechanisms that can be targeted for novel therapeutic approaches in stroke. Curr. Pharm. Des. 18, 3624–3644. 10.2174/138161212802002625 22574987 PMC3918413

[B132] Rosenberg G. A. Cunningham L. A. Wallace J. Alexander S. Estrada E. Y. Grossetete M.. (2001). Immunohistochemistry of matrix metalloproteinases in reperfusion injury to rat brain: activation of MMP-9 linked to stromelysin-1 and microglia in cell cultures. Brain Res. 893, 104–112. 10.1016/s0006-8993(00)03294-7 11222998

[B133] Ruhrberg C. Bautch V. L. (2013). Neurovascular development and links to disease. Cell. Mol. Life Sci. 70, 1675–1684. 10.1007/s00018-013-1277-5 23475065 PMC3632722

[B134] Ruhrberg C. Gerhardt H. Golding M. Watson R. Ioannidou S. Fujisawa H.. (2002). Spatially restricted patterning cues provided by heparin-binding VEGF-A control blood vessel branching morphogenesis. Genes Dev. 16, 2684–2698. 10.1101/gad.242002 12381667 PMC187458

[B135] Rymo S. F. Gerhardt H. Wolfhagen Sand F. Lang R. Uv A. Betsholtz C. (2011). A two-way communication between microglial cells and angiogenic sprouts regulates angiogenesis in aortic ring cultures. PLoS One 6:e15846. 10.1371/journal.pone.0015846 21264342 PMC3018482

[B136] Ryu J. K. McLarnon J. G. (2009). A leaky blood-brain barrier, fibrinogen infiltration and microglial reactivity in inflamed Alzheimer’s disease brain. J. Cell. Mol. Med. 13, 2911–2925. 10.1111/j.1582-4934.2008.00434.x 18657226 PMC4498946

[B137] Sandoval K. E. Witt K. A. (2008). Blood-brain barrier tight junction permeability and ischemic stroke. Neurobiol. Dis. 32, 200–219. 10.1016/j.nbd.2008.08.005 18790057

[B138] Schreibelt G. Kooij G. Reijerkerk A. van Doorn R. Gringhuis S. I. van der Pol S.. (2007). Reactive oxygen species alter brain endothelial tight junction dynamics via RhoA, PI3 kinase and PKB signaling. FASEB J. 21, 3666–3676. 10.1096/fj.07-8329com 17586731

[B139] Selvamani A. Sathyan P. Miranda R. C. Sohrabji F. (2012). An antagomir to microRNA Let7f promotes neuroprotection in an ischemic stroke model. PLoS One 7:e32662. 10.1371/journal.pone.0032662 22393433 PMC3290559

[B140] Sharief M. K. Ciardi M. Thompson E. J. (1992). Blood-brain barrier damage in patients with bacterial meningitis: association with tumor necrosis factor-alpha but not interleukin-1 *β*. J. Infect. Dis. 166, 350–358. 10.1093/infdis/166.2.350 1634806

[B141] Shechter R. Schwartz M. (2013). Harnessing monocyte-derived macrophages to control central nervous system pathologies: no longer ‘if’ but ‘how’. J. Pathol. 229, 332–346. 10.1002/path.4106 23007711

[B142] Sloka S. Metz L. M. Hader W. Starreveld Y. Yong V. W. (2013). Reduction of microglial activity in a model of multiple sclerosis by dipyridamole. J. Neuroinflammation 10:89. 10.1186/1742-2094-10-89 23866809 PMC3724584

[B143] Smith J. A. Das A. Ray S. K. Banik N. L. (2012). Role of pro-inflammatory cytokines released from microglia in neurodegenerative diseases. Brain Res. Bull. 87, 10–20. 10.1016/j.brainresbull.2011.10.004 22024597 PMC9827422

[B144] Sohrabji F. Williams M. (2013). Stroke neuroprotection: oestrogen and insulin-like growth factor-1 interactions and the role of microglia. J. Neuroendocrinol. 25, 1173–1181. 10.1111/jne.12059 23763366 PMC5630268

[B145] Spindler K. R. Hsu T. H. (2012). Viral disruption of the blood-brain barrier. Trends Microbiol. 20, 282–290. 10.1016/j.tim.2012.03.009 22564250 PMC3367119

[B146] Sriram S. Steiner I. (2005). Experimental allergic encephalomyelitis: a misleading model of multiple sclerosis. Ann. Neurol. 58, 939–945. 10.1002/ana.20743 16315280

[B148] Stamatovic S. M. Dimitrijevic O. B. Keep R. F. Andjelkovic A. V. (2006). Protein kinase Calpha-RhoA cross-talk in CCL2-induced alterations in brain endothelial permeability. J. Biol. Chem. 281, 8379–8388. 10.1074/jbc.m513122200 16439355

[B149] Stamatovic S. M. Keep R. F. Kunkel S. L. Andjelkovic A. V. (2003). Potential role of MCP-1 in endothelial cell tight junction ‘opening’: signaling via Rho and Rho kinase. J. Cell Sci. 116, 4615–4628. 10.1242/jcs.00755 14576355

[B150] Stamatovic S. M. Shakui P. Keep R. F. Moore B. B. Kunkel S. L. Van Rooijen N.. (2005). Monocyte chemoattractant protein-1 regulation of blood-brain barrier permeability. J. Cereb. Blood Flow Metab. 25, 593–606. 10.1038/sj.jcbfm.9600055 15689955

[B151] Stewart P. A. Wiley M. J. (1981). Developing nervous tissue induces formation of blood-brain barrier characteristics in invading endothelial cells: a study using quail—chick transplantation chimeras. Dev. Biol. 84, 183–192. 10.1016/0012-1606(81)90382-1 7250491

[B152] Strazza M. Pirrone V. Wigdahl B. Nonnemacher M. R. (2011). Breaking down the barrier: the effects of HIV-1 on the blood-brain barrier. Brain Res. 1399, 96–115. 10.1016/j.brainres.2011.05.015 21641584 PMC3139430

[B153] Sumi N. Nishioku T. Takata F. Matsumoto J. Watanabe T. Shuto H.. (2010). Lipopolysaccharide-activated microglia induce dysfunction of the blood-brain barrier in rat microvascular endothelial cells co-cultured with microglia. Cell. Mol. Neurobiol. 30, 247–253. 10.1007/s10571-009-9446-7 19728078 PMC11498813

[B154] Takeda S. Sato N. Morishita R. (2014). Systemic inflammation, blood-brain barrier vulnerability and cognitive/non-cognitive symptoms in Alzheimer disease: relevance to pathogenesis and therapy. Front. Aging Neurosci. 6:171. 10.3389/fnagi.2014.00171 25120476 PMC4114193

[B155] Tammela T. Zarkada G. Nurmi H. Jakobsson L. Heinolainen K. Tvorogov D.. (2011). VEGFR-3 controls tip to stalk conversion at vessel fusion sites by reinforcing Notch signalling. Nat. Cell Biol. 13, 1202–1213. 10.1038/ncb2331 21909098 PMC3261765

[B156] Tanaka S. Ide M. Shibutani T. Ohtaki H. Numazawa S. Shioda S.. (2006). Lipopolysaccharide-induced microglial activation induces learning and memory deficits without neuronal cell death in rats. J. Neurosci. Res. 83, 557–566. 10.1002/jnr.20752 16429444

[B157] Thornton P. McColl B. W. Greenhalgh A. Denes A. Allan S. M. Rothwell N. J. (2010). Platelet interleukin-1alpha drives cerebrovascular inflammation. Blood 115, 3632–3639. 10.1182/blood-2009-11-252643 20200351

[B158] Tikka T. Fiebich B. L. Goldsteins G. Keinanen R. Koistinaho J. (2001). Minocycline, a tetracycline derivative, is neuroprotective against excitotoxicity by inhibiting activation and proliferation of microglia. J. Neurosci. 21, 2580–2588. 11306611 10.1523/JNEUROSCI.21-08-02580.2001 PMC6762519

[B159] Town T. Tan J. Flavell R. A. Mullan M. (2005). T-cells in Alzheimer’s disease. Neuromolecular Med. 7, 255–264. 10.1385/NMM:7:3:255 16247185

[B160] Truettner J. S. Alonso O. F. Dalton Dietrich W. (2005). Influence of therapeutic hypothermia on matrix metalloproteinase activity after traumatic brain injury in rats. J. Cereb. Blood Flow Metab. 25, 1505–1516. 10.1038/sj.jcbfm.9600150 15959464

[B161] Tu Y. F. Tsai Y. S. Wang L. W. Wu H. C. Huang C. C. Ho C. J. (2011). Overweight worsens apoptosis, neuroinflammation and blood-brain barrier damage after hypoxic ischemia in neonatal brain through JNK hyperactivation. J. Neuroinflammation 8:40. 10.1186/1742-2094-8-40 21518436 PMC3090337

[B162] Ubenauf K. M. Krueger M. Henneke P. Berner R. (2007). Lipopolysaccharide binding protein is a potential marker for invasive bacterial infections in children. Pediatr. Infect. Dis. J. 26, 159–162. 10.1097/01.inf.0000253064.88722.6d 17259880

[B163] van Horssen J. Vos C. M. Admiraal L. van Haastert E. S. Montagne L. van der Valk P.. (2006). Matrix metalloproteinase-19 is highly expressed in active multiple sclerosis lesions. Neuropathol. Appl. Neurobiol. 32, 585–593. 10.1111/j.1365-2990.2006.00766.x 17083473

[B164] Verderio C. Matteoli M. (2001). ATP mediates calcium signaling between astrocytes and microglial cells: modulation by IFN-gamma. J. Immunol. 166, 6383–6391. 10.4049/jimmunol.166.10.6383 11342663

[B165] Vezzani A. French J. Bartfai T. Baram T. Z. (2011). The role of inflammation in epilepsy. Nat. Rev. Neurol. 7, 31–40. 10.1038/nrneurol.2010.178 21135885 PMC3378051

[B166] Vezzani A. Ravizza T. Balosso S. Aronica E. (2008). Glia as a source of cytokines: implications for neuronal excitability and survival. Epilepsia 49(Suppl. 2), 24–32. 10.1111/j.1528-1167.2008.01490.x 18226169

[B167] Wang L. Lim E. J. Toborek M. Hennig B. (2008). The role of fatty acids and caveolin-1 in tumor necrosis factor alpha-induced endothelial cell activation. Metabolism 57, 1328–1339. 10.1016/j.metabol.2008.01.036 18803934 PMC3349996

[B168] Wang L. W. Tu Y. F. Huang C. C. Ho C. J. (2012). JNK signaling is the shared pathway linking neuroinflammation, blood-brain barrier disruption and oligodendroglial apoptosis in the white matter injury of the immature brain. J. Neuroinflammation 9:175. 10.1186/1742-2094-9-175 22805152 PMC3414763

[B169] Weaver A. Goncalves da Silva A. Nuttall R. K. Edwards D. R. Shapiro S. D. Rivest S.. (2005). An elevated matrix metalloproteinase (MMP) in an animal model of multiple sclerosis is protective by affecting Th1/Th2 polarization. FASEB J. 19, 1668–1670. 10.1096/fj.04-2030fje 16081501

[B170] Wiley C. A. Schrier R. D. Nelson J. A. Lampert P. W. Oldstone M. B. (1986). Cellular localization of human immunodeficiency virus infection within the brains of acquired immune deficiency syndrome patients. Proc. Natl. Acad. Sci. U S A 83, 7089–7093. 10.1073/pnas.83.18.7089 3018755 PMC386658

[B171] Willis C. L. (2011). Glia-induced reversible disruption of blood-brain barrier integrity and neuropathological response of the neurovascular unit. Toxicol. Pathol. 39, 172–185. 10.1177/0192623310385830 21189317

[B172] Witt K. A. Mark K. S. Huber J. Davis T. P. (2005). Hypoxia-inducible factor and nuclear factor kappa-B activation in blood-brain barrier endothelium under hypoxic/reoxygenation stress. J. Neurochem. 92, 203–214. 10.1111/j.1471-4159.2004.02871.x 15606909

[B173] Wolburg H. Lippoldt A. (2002). Tight junctions of the blood-brain barrier: development, composition and regulation. Vascul. Pharmacol. 38, 323–337. 10.1016/s1537-1891(02)00200-8 12529927

[B174] Wyss-Coray T. (2006). Tgf-Beta pathway as a potential target in neurodegeneration and Alzheimer’s. Curr. Alzheimer Res. 3, 191–195. 10.2174/156720506777632916 16842094

[B175] Xiong Y. Zhang Y. Mahmood A. Meng Y. Zhang Z. G. Morris D. C.. (2012). Neuroprotective and neurorestorative effects of thymosin beta4 treatment initiated 6 hours after traumatic brain injury in rats. J. Neurosurg. 116, 1081–1092. 10.3171/2012.1.jns111729 22324420 PMC3392183

[B176] Yamagata K. Tagami M. Takenaga F. Yamori Y. Itoh S. (2004). Hypoxia-induced changes in tight junction permeability of brain capillary endothelial cells are associated with IL-1beta and nitric oxide. Neurobiol. Dis. 17, 491–499. 10.1016/j.nbd.2004.08.001 15571984

[B177] Yang Y. Estrada E. Y. Thompson J. F. Liu W. Rosenberg G. A. (2007). Matrix metalloproteinase-mediated disruption of tight junction proteins in cerebral vessels is reversed by synthetic matrix metalloproteinase inhibitor in focal ischemia in rat. J. Cereb. Blood Flow Metab. 27, 697–709. 10.1038/sj.jcbfm.9600375 16850029

[B178] Yang Y. Rosenberg G. A. (2011). Blood-brain barrier breakdown in acute and chronic cerebrovascular disease. Stroke 42, 3323–3328. 10.1161/strokeaha.110.608257 21940972 PMC3584169

[B179] Yang Y. M. Shang D. S. Zhao W. D. Fang W. G. Chen Y. H. (2013). Microglial TNF-alpha-dependent elevation of MHC class I expression on brain endothelium induced by amyloid-beta promotes T cell transendothelial migration. Neurochem. Res. 38, 2295–2304. 10.1007/s11064-013-1138-5 23990225

[B180] Yao Y. Tsirka S. E. (2011). Truncation of monocyte chemoattractant protein 1 by plasmin promotes blood-brain barrier disruption. J. Cell Sci. 124, 1486–1495. 10.1242/jcs.082834 21486949 PMC3078815

[B181] Yenari M. A. Xu L. Tang X. N. Qiao Y. Giffard R. G. (2006). Microglia potentiate damage to blood-brain barrier constituents: improvement by minocycline in vivo and in vitro. Stroke 37, 1087–1093. 10.1161/01.str.0000206281.77178.ac 16497985

[B182] Yin X. Wright J. Wall T. Grammas P. (2010). Brain endothelial cells synthesize neurotoxic thrombin in Alzheimer’s disease. Am. J. Pathol. 176, 1600–1606. 10.2353/ajpath.2010.090406 20150433 PMC2843451

[B183] Yrjänheikki J. Keinänen R. Pellikka M. Hökfelt T. Koistinaho J. (1998). Tetracyclines inhibit microglial activation and are neuroprotective in global brain ischemia. Proc. Natl. Acad. Sci. U S A 95, 15769–15774. 10.1073/pnas.95.26.15769 9861045 PMC28119

[B184] Yrjänheikki J. Tikka T. Keinänen R. Goldsteins G. Chan P. H. Koistinaho J. (1999). A tetracycline derivative, minocycline, reduces inflammation and protects against focal cerebral ischemia with a wide therapeutic window. Proc. Natl. Acad. Sci. U S A 96, 13496–13500. 10.1073/pnas.96.23.13496 10557349 PMC23976

[B185] Zhou X. Spittau B. Krieglstein K. (2012). TGFbeta signalling plays an important role in IL4-induced alternative activation of microglia. J. Neuroinflammation 9:210. 10.1186/1742-2094-9-210 22947253 PMC3488564

[B186] Zipser B. D. Johanson C. E. Gonzalez L. Berzin T. M. Tavares R. Hulette C. M.. (2007). Microvascular injury and blood-brain barrier leakage in Alzheimer’s disease. Neurobiol. Aging 28, 977–986. 10.1016/j.neurobiolaging.2006.05.016 16782234

[B187] Zlokovic B. V. (2004). Clearing amyloid through the blood-brain barrier. J. Neurochem. 89, 807–811. 10.1111/j.1471-4159.2004.02385.x 15140180

[B188] Zlokovic B. V. (2008). The blood-brain barrier in health and chronic neurodegenerative disorders. Neuron 57, 178–201. 10.1016/j.neuron.2008.01.003 18215617

[B189] Zlokovic B. V. (2011). Neurovascular pathways to neurodegeneration in Alzheimer’s disease and other disorders. Nat. Rev. Neurosci. 12, 723–738. 10.1038/nrn3114 22048062 PMC4036520

